# Identifying the EMT-related signature to stratify prognosis and evaluate the tumor microenvironment in lung adenocarcinoma

**DOI:** 10.3389/fgene.2022.1008416

**Published:** 2022-09-16

**Authors:** Feng Li, Qing-Zhen Song, Yi-Fan Zhang, Xing-Ru Wang, Li-Min Cao, Nan Li, Ling-Xia Zhao, Sheng-Xiao Zhang, Xiao-Fei Zhuang

**Affiliations:** ^1^ Department of Cell Biology, Shanxi Province Cancer Hospital, Shanxi Hospital Affiliated to Cancer Hospital, Chinese Academy of Medical Sciences, Cancer Hospital Affiliated to Shanxi Medical University, Taiyuan, China; ^2^ Department of Special Geriatrics, Shanxi Province Cancer Hospital, Shanxi Hospital Affiliated to Cancer Hospital, Chinese Academy of Medical Sciences, Cancer Hospital Affiliated to Shanxi Medical University, Taiyuan, China; ^3^ The First Clinical Medical College, Shanxi Medical University, Taiyuan, China; ^4^ Key Laboratory of Cellular Physiology at Shanxi Medical University, Ministry of Education, Taiyuan, China; ^5^ The Second Clinical Medical College, Shanxi Medical University, Taiyuan, China; ^6^ The School of Basic Medicine of Shanxi Medical University, Taiyuan, Shanxi, China; ^7^ Department of Endocrinology and Metabolism, Shanxi Bethune Hospital, Shanxi Academy of Medical Sciences, Tongji Shanxi Hospital, Third Hospital of Shanxi Medical University, Taiyuan, China; ^8^ Department of Rheumatology, The Second Hospital of Shanxi Medical University, Taiyuan, Shanxi, China; ^9^ Department of Thoracic Surgery, Shanxi Province Cancer Hospital, Shanxi Hospital Affiliated to Cancer Hospital, Chinese Academy of Medical Sciences, Cancer Hospital Affiliated to Shanxi Medical University, Taiyuan, China

**Keywords:** epithelial-mesenchymal transition, lung adenocarcinoma, prognosis, tumor immune microenvironment, immunotherapy, chemotherapy

## Abstract

**Background:** Epithelial-mesenchymal transition (EMT) is a critical process in tumor invasion and metastasis. EMT has been shown to significantly influence the invasion, metastasis, and poor prognosis in lung adenocarcinoma (LUAD). This study aimed to develop a novel EMT-related prognostic model capable of predicting overall survival (OS) in patients with LUAD.

**Methods:** A total of *283* LUAD patients from TCGA RNA-seq dataset were assigned to a training cohort for model building, and 310 LUAD patients from GEO RNA-seq dataset were assigned to a validation cohort. EMT genes were acquired from MsigDB database and then prognosis-related EMT genes were identified by univariate Cox regression. Lasso regression was then performed to determine the genes and the corresponding variables to construct a prognosis risk model from the training cohort. Furthermore, characteristics of the tumor microenvironment (TME), mutation status and chemotherapy responses were analyzed to assess the differences between the two risk groups based on the prognostic model. In addition, RT-qPCR was employed to validate the expression patterns of the 6 genes derived from the risk model.

**Results:** A six-gene EMT signature (PMEPA1, LOXL2, PLOD2, MMP14, SPOCK1 and DCN) was successfully constructed and validated. The signature assigned the LUAD patients into high-risk and low-risk groups. In comparison with the low-risk group, patients in the high-risk group had a significantly lower survival rate. ROC curves and calibration curves for the risk model demonstrated reliable stratification and predictive ability. The risk model was robustly correlated with multiple TME characteristics. Besides, the data showed that patients in the low-risk group had more immune activities, higher stemness scores and cytolytic activity scores and higher TMB. In addition, RT-qPCR results revealed that PMEPA1, LOXL2, PLOD2, MMP14, and SPOCK1 were notably upregulated in LUAD tissues, while DCN was downregulated.

**Conclusion:** Our study successfully developed a novel EMT-related signature to predict prognosis of LUAD patients and guide treatment strategies. The six genes derived from the prediction signature might play a potential role in antitumor immunity and serve as promising therapeutic targets in LUAD.

## Introduction

Lung cancer is the leading cause of death from cancer worldwide, which contributed to approximately 1,800,000 deaths in 2020 (Sung et al., 2021). The majority of the lung cancers are lung adenocarcinoma (LUAD), which are highly invasive, with a rapid metastatic spread, highlighting the systemic threat of the disease (Devarakonda et al., 2015).

In the past two decades, there have been important advances in characterization of mutational spectrum and molecular subtypes of LUAD, which have led to development of targeted therapies resulting in dramatically improved patient outcomes (Rotow and Bivona, 2017). However, clinical application in targeting RAS signaling or rescuing the tumor suppressor TP53 gene, which have been recommended as LUAD therapies, remains challenging (Kim et al., 2021). Besides, treatment regimens that target epidermal growth factor receptor (EGFR) and anaplastic lymphoma kinase have only benefitted a small percentage of LUAD patients (Wang Q. et al., 2020). Hence, there is an urgent need for identification of prognostic biomarkers as well as effective drug targets.

Spread of cancer cells due to metastasis is the leading cause of death in patients with primary lung cancer (Prateep et al., 2018). Epithelial-mesenchymal transition (EMT) is an important mechanism driving the tumor metastasis process, where epithelial cells lose their morphology and subsequently change to mesenchymal phenotypes, thereby acquiring features of mesenchymal cells such as motility and invasiveness (Iwatsuki et al., 2010). Loss of E-cadherin is a hallmark of EMT, which leads to decreased intercellular adhesion and enhanced cell motility (Thiery et al., 2009). Previous data have suggested that EMT is associated with neoplastic aggressiveness and progression in various malignancies including LUAD.

Tumor microenvironment (TME) is comprised of stromal and immune cells that secrete a variety of cytokines, chemokines and growth factors, which have been shown to induce EMT in nearby cancer cells through direct activation of various EMT-induced transcription factors (EMT-TFs) or inhibition of expression of effector molecules that promote mesenchymal cell state (Gupta and Massagué, 2006; Grivennikov et al., 2010). Besides, EMT has also been reported to play a vital role in tumor malignancy, immune regulation and initiation of therapeutic responses by inducing cell phenotypic plasticity, inflammatory, and immunosuppressive TME, leading to resistance to immunotherapy and chemotherapy (Terry et al., 2017). In addition, EMT status was associated with the activation of varied immune checkpoint molecules (Datar and Schalper, 2016). Therefore, it is important to understand the underlying mechanisms mediating the interaction between EMT and TME. The development of an EMT-related signature may contribute to the provision of potential biomarkers for LUAD and help enhance the understanding of the immunogenomic profile of LUAD.

In this study, we developed an EMT-related signature (ERGS) related to prognosis based on The Cancer Genome Atlas (TCGA) database which was validated by the Gene Expression Omnibus (GEO) database. The results demonstrated that LUAD patients with high-risk scores were strongly associated with shorter overall survival (OS) compared with patients in the high low-risk score group. We then explored the difference in immune infiltration and mutation landscape between the two risk groups and analyzed patients’ response to the immune checkpoint inhibitor (ICI) therapy and chemotherapy. Together, the high-risk group was more likely to experience immunosuppression, thus less likely to benefit from either of the treatment options, which is consistent with the EMT features. In a nutshell, our study highlights a functional role of the ERGs and uncovers a potential prognostic biomarker for individualized treatment of LUAD.

## Methods

### Collection of the clinical samples

A total of 10 pairs of LUAD tissues and adjacent non-tumor tissues were collected from patients who received surgical resection at Shanxi Cancer Hospital (Shanxi, China) from January to September 2021. The patients were not subjected to any anti-cancer treatment before surgery. Tissue specimens were frozen in liquid nitrogen within 30 min of resection and stored at –80°C for analysis. Our study was approved by the ethics committee of Shanxi Cancer Hospital (sxszl-F-375), and was conducted in accordance with the principles of Declaration of Helsinki.

### Dataset acquisition and processing

The RNA-sequencing data and corresponding clinical information of 341 samples in the TCGA-LUAD cohort were obtained from the UCSC Cancer Genomics Browser database (https://genome-cancer.ucsc.edu/) and used as the training cohort, which included 283 tumor samples and 58 tumor-adjacent tissue samples. Another 310 LUAD samples in the GSE72094 from the GEO database (https://www.ncbi.nlm.nih.gov/geo/) were used as an external validation cohort. Gene expression profiles were all quantified with fragments per kilobase of transcript per million mapped reads (FPKM) and normalized using log2-based transformations. Because the data from TCGA and GEO datasets are publicly available, there was no requirement for institutional review board approval and informed consent from the patients. EMT-related genes (ERGs) from the gene set “HALLMARK_EPITHELIAL_MESENCHYMAL_TRANSITION” were downloaded from GSEA (https://www.gsea-msigdb.org/gsea/index.jsp) as shown in Supplementary Table S1.

### Identification of differentially expressed ERGs

The “limma” package in R software was used to identify differentially expressed genes (DEGs) between LUAD and tumor-adjacent tissue samples, where |log2FC| > 0.32 and FDR < 0.05 were set as filters. The Venn diagram was generated using the “Venn Diagram” R package to identify DEGs related to the EMT process. The DE-ERGs were visualized as volcano plots and heat maps by “pheatmap” and “ggplot2” R packages, respectively.

### Construction and establishment of the ERG prognostic signature

Univariate Cox regression analysis was employed to determine the DE-ERGs associated with OS of the patients. The DE-ERGs most related to OS with a *p* < 0.05 were further picked out for least absolute shrinkage and selector operator (LASSO) Cox regression. The analysis narrowed down the candidate DE-ERGs which were used to construct a prognostic model. To find optimal penalization terms, a penalty regularization parameter (λ) was determined by ten-fold cross validation following the minimum criteria (i.e., the value of λ corresponding to the lowest partial likelihood deviance). The risk score of each patient was calculated based on the normalized expression level of each gene in the prognostic signature and its relevant regression coefficients. The formula of the model was: Risk score = ∑(expression of signature genes × corresponding coefficient). Based on the median of the risk score, LUAD patients were assigned into high- or low-risk groups.

### Evaluation and validation of the ERG prognostic signature

To determine the potential value of the ERG prognostic signature in predicting the prognosis in LUAD patients, Kaplan-Meier survival curves were utilized to assess the prognostic value in the high- and low-risk groups using the “survminer” R package. The predictive ability of the ERG prognostic signature was assessed using the AUC values of the time-dependent receiver operating characteristic (ROC) curves generated by the “pROC” package. To explore the distribution of different risk groups, we performed t-SNE for cluster visualization using the “Rtsne” R package. In addition, univariate and multivariate Cox analyses were conducted to determine whether the risk score was independent from other clinicopathological characteristics (age, gender, T stage, N status and smoking status). Thereafter, the ERG prognostic signature was validated in an independent cohort obtained from the GEO database.

### Construction of the nomogram

Based on all independent prognostic factors determined by the multivariate analysis, we established a nomogram to predict 1-, 3-, and 5-years OS of LUAD patients using the “rms” R package. The concordance index (C-index) was calculated to appraise the discriminative ability of the nomogram while the calibration curve was performed to evaluate the accuracy of the nomogram.

### Mutation analysis

The “TCGAbiolinks” R package was used to download the somatic mutation profiles based on the segment mean value log2 (copy-number/2) of LUAD patients from the TCGA cohort, while the “maftools” package was employed for analysis and visualization of the data. We then used Oncoplot to display the top 20 genes with high mutation frequency in LUAD patient samples in high- and low-risk groups. Besides, the tumor mutation burden (TMB) was calculated as the total number of somatic, coding, indel mutations and base substitution for each mega-base of the genome under analysis. The correlation between TMB levels and risk score was analyzed using the Spearman’s correlation. In addition, we assessed the effect of the risk score combined with the TMB on the survival of LUAD patients.

### Analysis of tumor immune microenvironment

To identify the immune infiltration characteristics in LUAD, the 22 immune cells from each LUAD sample from the TCGA cohort was quantified based on standardized gene expression profile using the CIBERSORT algorithm. The “ESTIMATE” R package was employed to compute the stromal score, immune score, and estimate score. As a measure of inflammation, the cytolytic activity (CYT) score was computed as the geometric mean of the RPKM expression of granzyme A (GZMA) and perforin-1 (PRF1) mRNA expression levels in the tumor tissues (Wakiyama et al., 2018). We also computed mRNA stemness index (mRNAsi) using the “TCGAbiolinks” R package based on the mRNA levels obtained from one-class logistic regression machine learning (OCLR) algorithm (Guo et al., 2021), where a higher mRNAsi represents a greater tumor dedifferentiation and higher cancer stem cell levels.

### Assessment of immunotherapy response

Tumor Immune Dysfunction and Exclusion (TIDE) algorithm was employed to evaluate the likelihood of each sample to respond to immunotherapy. TIDE algorithm is a computational method used to model two primary mechanisms of tumor immune evasion: the induction of T cell dysfunction in tumors with high infiltration of cytotoxic T lymphocytes (CTL) and prevention of T cell infiltration in tumors with low CTL level. Immune checkpoint expression has a significant impact on the immunotherapy treatment responses. To further investigate the influence of the ERG scores on immunotherapy, the differential expression of immune checkpoint-related genes between the two ERG subgroups were analyzed.

### Estimation of chemotherapy response

We also predicted the chemotherapy response of each LUAD patient based on information obtained from the Genomics of Drug Sensitivity in Cancer (GDSC) database. Four common chemotherapeutic agents (cisplatin, paclitaxel, gemcitabine, and docetaxel) and two small molecule inhibitors targeting EGFR (erlotinib and gefitinib) were selected and used at default parameters, which are approved in the treatment of LUAD cases. The prediction procedure was conducted using the “pRRophetic” R package where sensitivity to the drug was quantified by half-maximal inhibitory concentration (IC50) predicted through ridge regression. A low IC50 indicates that the patients are more sensitive to the drug.

### Gene set enrichment analysis

For further exploration of differences in biological pathways between high-risk and low-risk groups, GSEA was performed to assess Gene Ontology (GO) and the Kyoto Encyclopedia of Genes and Genomes (KEGG) using “clusterProfiler” R package.

### Reverse transcription-quantitative PCR

RT-qPCR was employed to quantify the expression of genes in ERGS in clinical specimens. We extracted total RNA from the LUAD and adjacent non-tumor tissues using the TRIzol reagent (Invitrogen, CA, United States). We synthesized cDNA from the total mRNA using PrimeScriptTM RT Master Mix (RR036B, Takara). Quantitative PCR was performed to analyze the mRNA expression levels of the ERGs genes using GoTaq® qPCR Master Mix (Promega, A6001). The RT-qPCR was performed in ABI Vii7 Sequence detection system (ABI, United States). We then compared the mRNA expression levels of PMEPA1, LOXL2, PLOD2, MMP14, SPOCK1, and DCN using the 2-ΔΔCT method. The primer sequences are shown in Table 1.

**TABLE 1:** Primers used in RT-qPCR.

Gene	Primer sequences (5′-3′)
PMEPA1	FORWARD CGT​AGG​TGA​AAA​GGC​AGA​ACA
	REVERSE GAC​ACA​GCT​CAA​CAA​AGA​AAC​GT
LOXL2	FORWARD ACA​GAA​TGT​GAA​GGA​GAC​ATC​C
	REVERSE TGA​TGT​TGT​TGG​AGT​AAT​CGG​A
PLOD2	FORWARD GGA​TGC​AGA​TGT​TGT​TTT​GAC​A
	REVERSE GCT​TTC​CAT​GAC​GAG​TTA​CAA​G
MMP14	FORWARD CAA​GAT​TGA​TGC​TGC​TCT​CTT​C
	REVERSE ACT​TTG​ATG​TTC​TTG​GGG​TAC​T
SPOCK1	FORWARD CAG​AAA​CTG​GAA​TCC​CAA​CAA​G
	REVERSE TTG​CAC​TTG​ACC​AAA​TTC​GAA​G
DCN	FORWARD GAC​AAC​AAC​AAG​CTT​ACC​AGA​G
	REVERSE TGA​AAA​GAC​TCA​CAC​CCG​AAT​A
GAPDH	FORWARD TGA​CTT​CAA​CAG​CGA​CAC​CCA
	REVERSE CAC​CCT​GTT​GCT​GTA​GCC​AAA

### Immunohistochemical analysis

To further validate the expression f the signature genes, we analyzed immunohistochemistry (IHC) staining data of ERG proteins in lung cancer and normal lung tissues from the Human Protein Atlas (HPA) online database (https://www.proteinatlas.org/).

### Statistical analysis

All statistical analyses and visualization were performed using R version 4.1.3. Differences between two groups were compared via the Wilcoxon rank-sum test or Kruskal-Wallis test in cases where the data did not follow normal distribution and the variance was unknown. The survival difference was evaluated using log-rank tests. In addition, correlation analyses between two continuous variables were evaluated by Spearman rank correlation test while K-nearest neighbor (k-NN) imputation was performed to impute the missing AUC values. A P value of less than 0.05 (two-sided) was considered statistically significant.

## Results

### Patient characteristics

**FIGURE 1:**
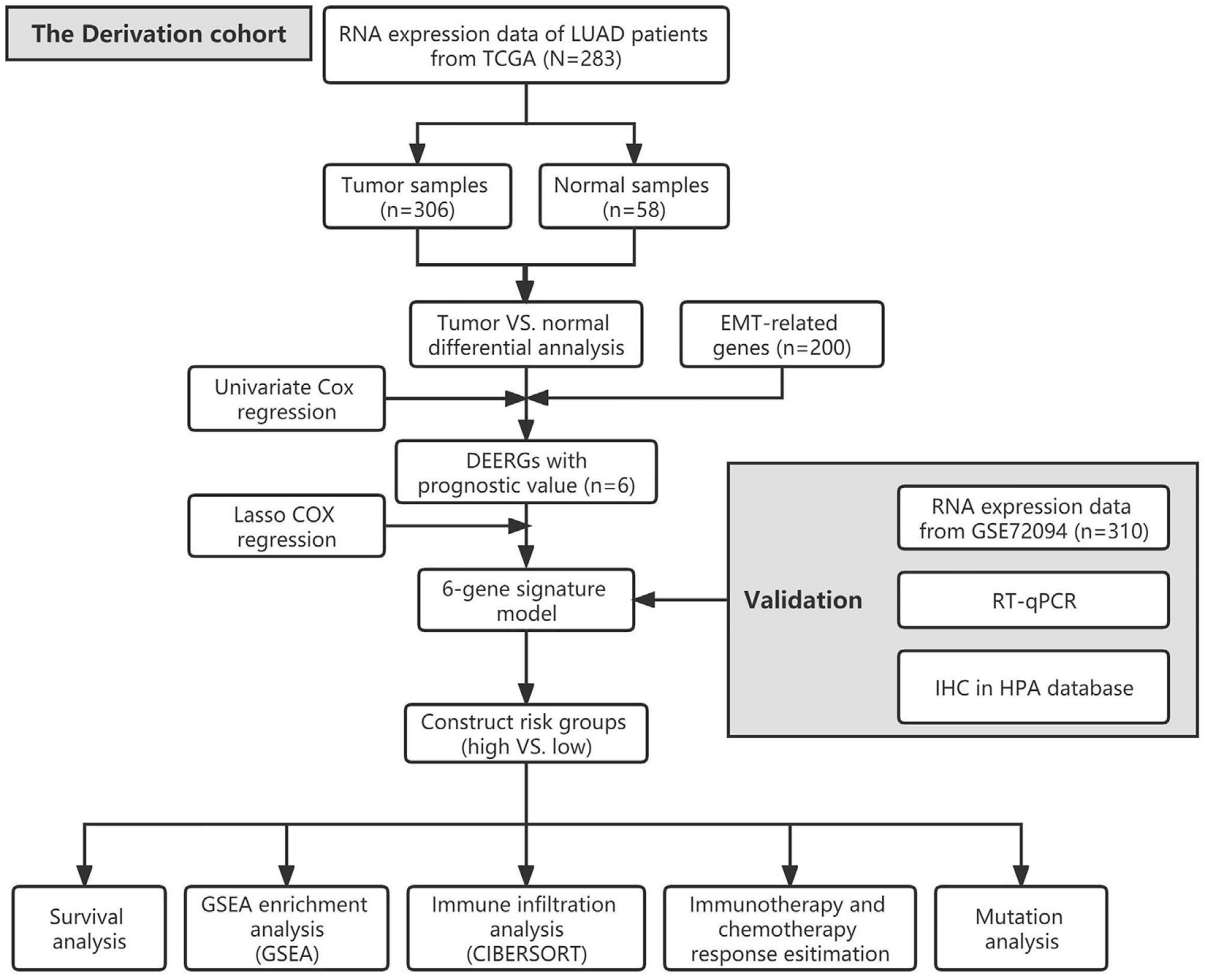
The flow chart of our study.

The flow chart of our study is shown in Figure 1. A total of 341 LUAD patients from the TCGA cohort were defined as a training set, while 310 patients from the GSE72094 cohort were used for external validation. The detailed clinical information of these patients is as summarized in Supplementary Table S2.

### Identification of differentially expressed ERGs

**FIGURE 2:**
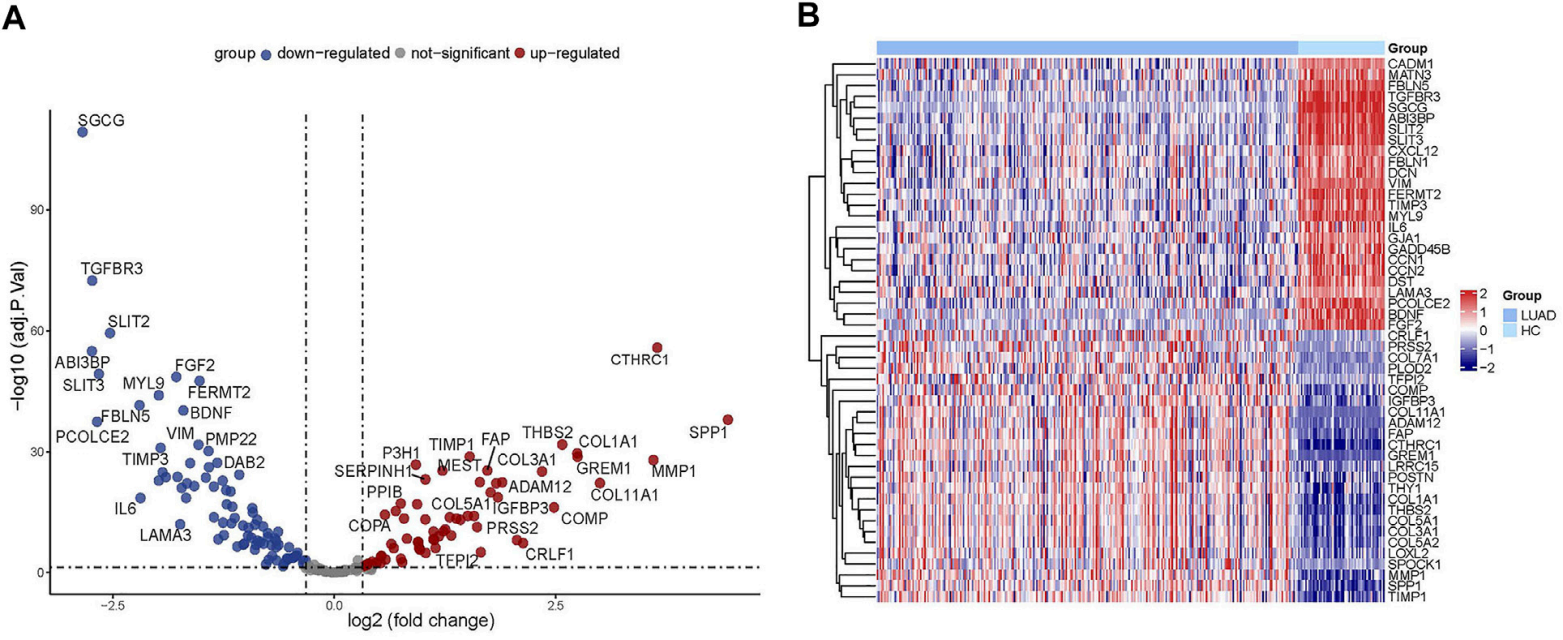
Analysis of differentially expressed ERGs. **(A)** Volcano plot showing the downregulated and upregulated ERGs. **(B)** Heatmap of the differentially expressed ERGs in LUAD.

Through the differential gene screening analysis, we retrieved 149 differentially expressed ERGs, which included 83 downregulated and 66 upregulated genes as shown in Figure 2A. The expression of the ERGs in LUAD samples and normal samples was displayed in a heat map (Figure 2B).

### Construction and assessment of ERGs

**FIGURE 3:**
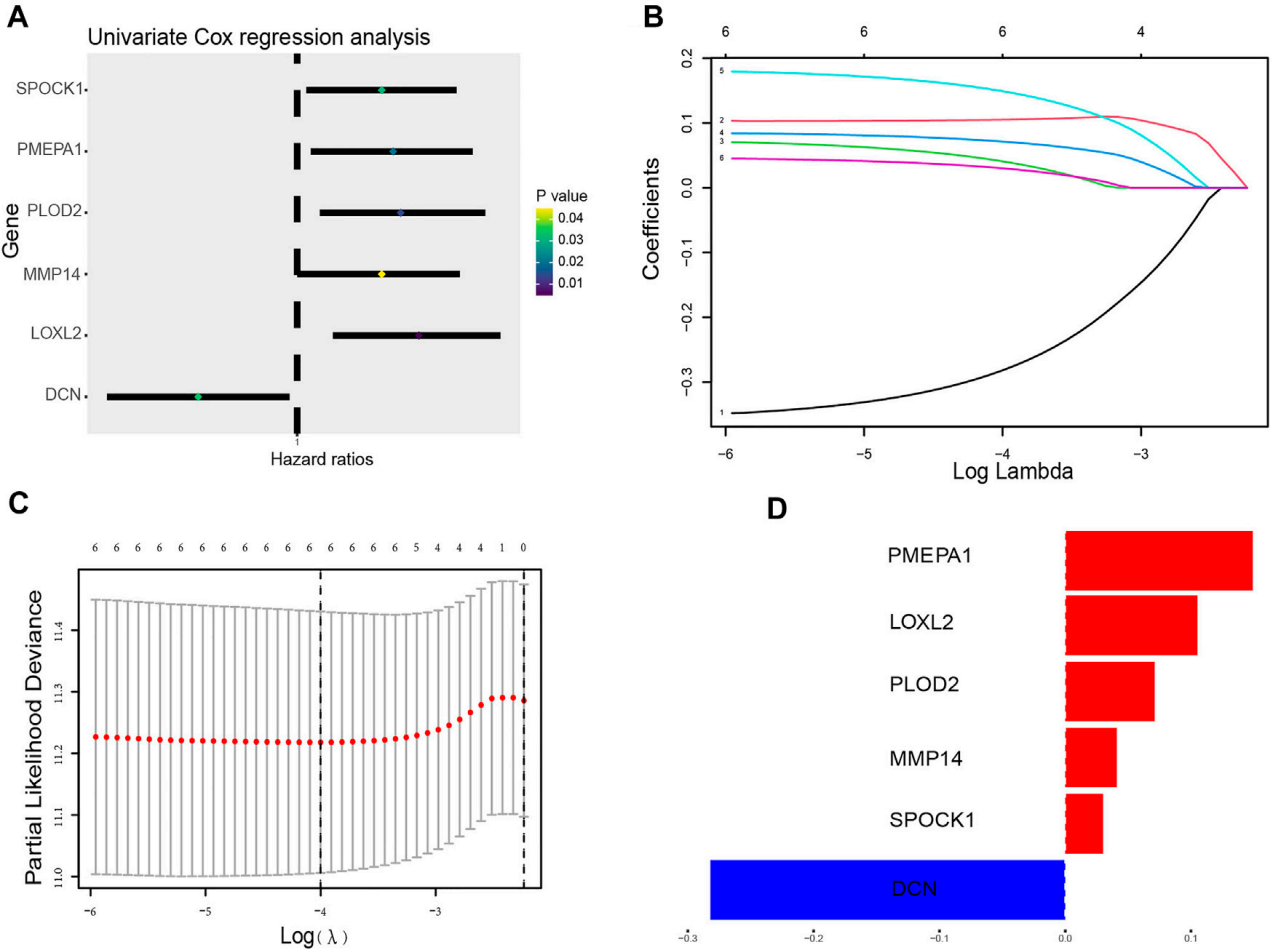
Construction of the ERG prognostic signature. **(A)** The forest plots illustrate univariate Cox analysis of the six genes significantly associated with OS. **(B)** Ten-time cross-validation for tuning parameter selection in the LASSO model. **(C)** LASSO coefficient profiles of the six ERGs significantly associated with OS. **(D)** The expression level of the 6 genes identified by Lasso regression analysis.

**FIGURE 4:**
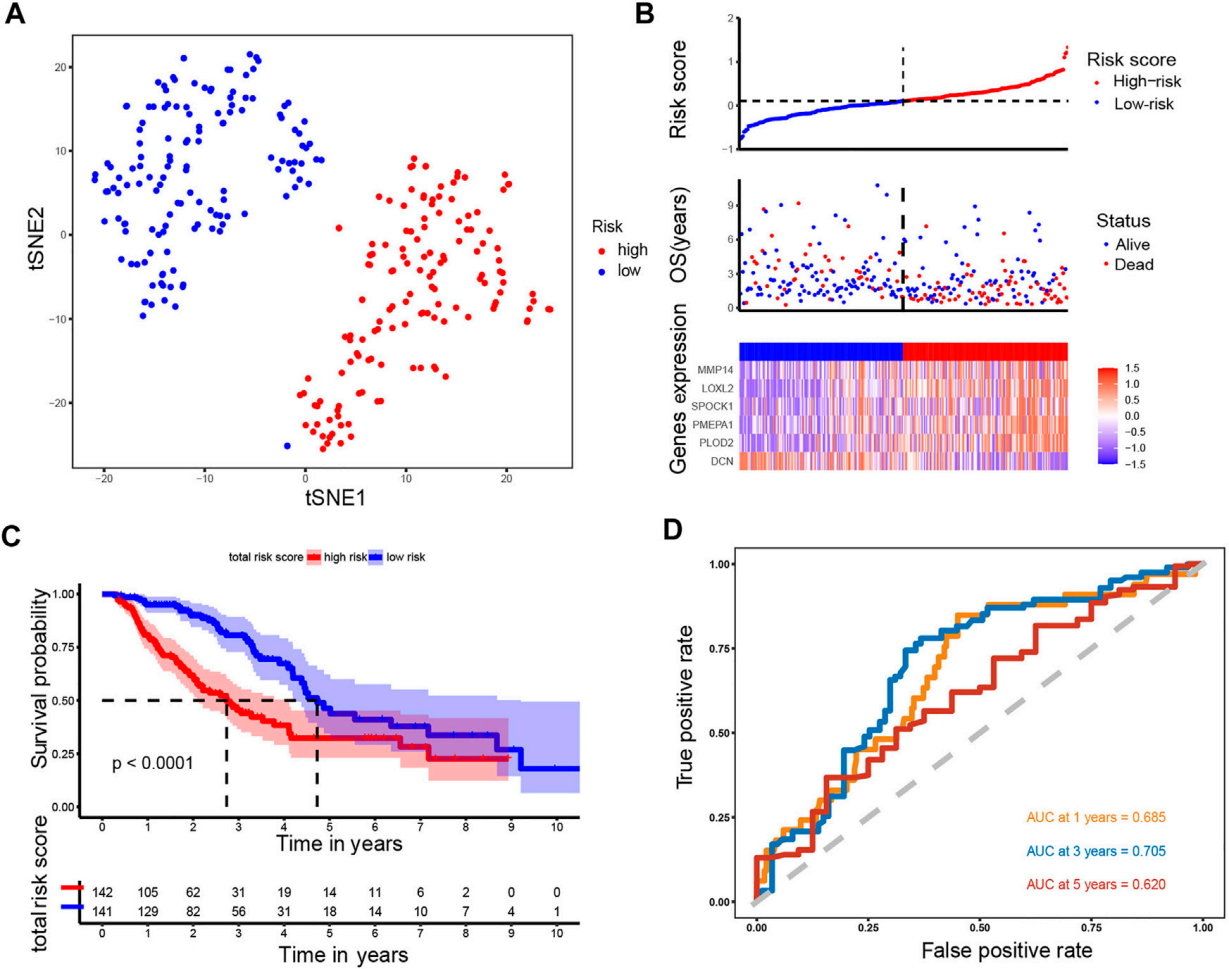
Analysis of the prognostic model in the training set. **(A)** t-SNE was used to evaluate whether the samples could be grouped correctly based on the ERGs risk score. **(B)** Heatmap showing the expression of six crucial genes in high- and low-risk groups and the distribution of risk scores and survival status of the LUAD patients with increasing risk score. **(C)** KM survival analysis between the high- and low-risk groups. **(D)** ROC curves analysis of the ERGs on OS at 1 year, 3 years, and 5 years.

To establish a prognostic model, Cox regression and LASSO regression were performed in the training set. First, we employed univariate Cox proportional hazard regression to identify prognosis-related genes from 149 DE-ERGs. Using a *p* value <0.05, 6 prognosis-related genes were identified (Figure 3A) (Supplementary Table S3). The 6 prognosis-related genes were further included in LASSO Cox regression based on the optimal value of λ to eliminate overfit genes and narrow down the range of model genes (Figures 3B,C) (Supplementary Table S4). After the LASSO regression analysis, 6 ERGs were used to construct the prognostic model consisting of PMEPA1, LOXL2, PLOD2, MMP14, SPOCK1 and DCN genes (Figure 3D). According to the coefficients and standardized expression of the six genes, the risk score of each LUAD patient from the TCGA dataset was calculated as follows: Risk Score = (−0.282*DCN) + (0.105*LOXL2) + (0.041*MMP14) + (0.071*PLOD2) + (0.149*PMEPA1) + (0.03*SPOCK1) (Supplementary Table S5). Taking the median risk score of 0.104 as cut-off, the patients were divided into high- (N = 142) and low-risk groups (N = 141). The t-SNE analysis revealed that the patients in different risk groups were distributed in two directions (Figure 4A). We defined the risk scores rank distribution, survival status and expression patterns of the 6 ERGs in LUAD patients (Figure 4B). Besides, the Kaplan-Meier survival analysis demonstrated that patients in the high-risk group had significantly poorer OS compared to those in the low-risk group (*p* < 0.001) (Figure 4C). In addition, as depicted in Figure 4D, the AUC value of the ROC curves for 1-year, 3-year, and 5-year OS was 0.685, 0.705 and 0.620, respectively, in the TCGA cohort.

### Validation of the prognostic signature

**FIGURE 5:**
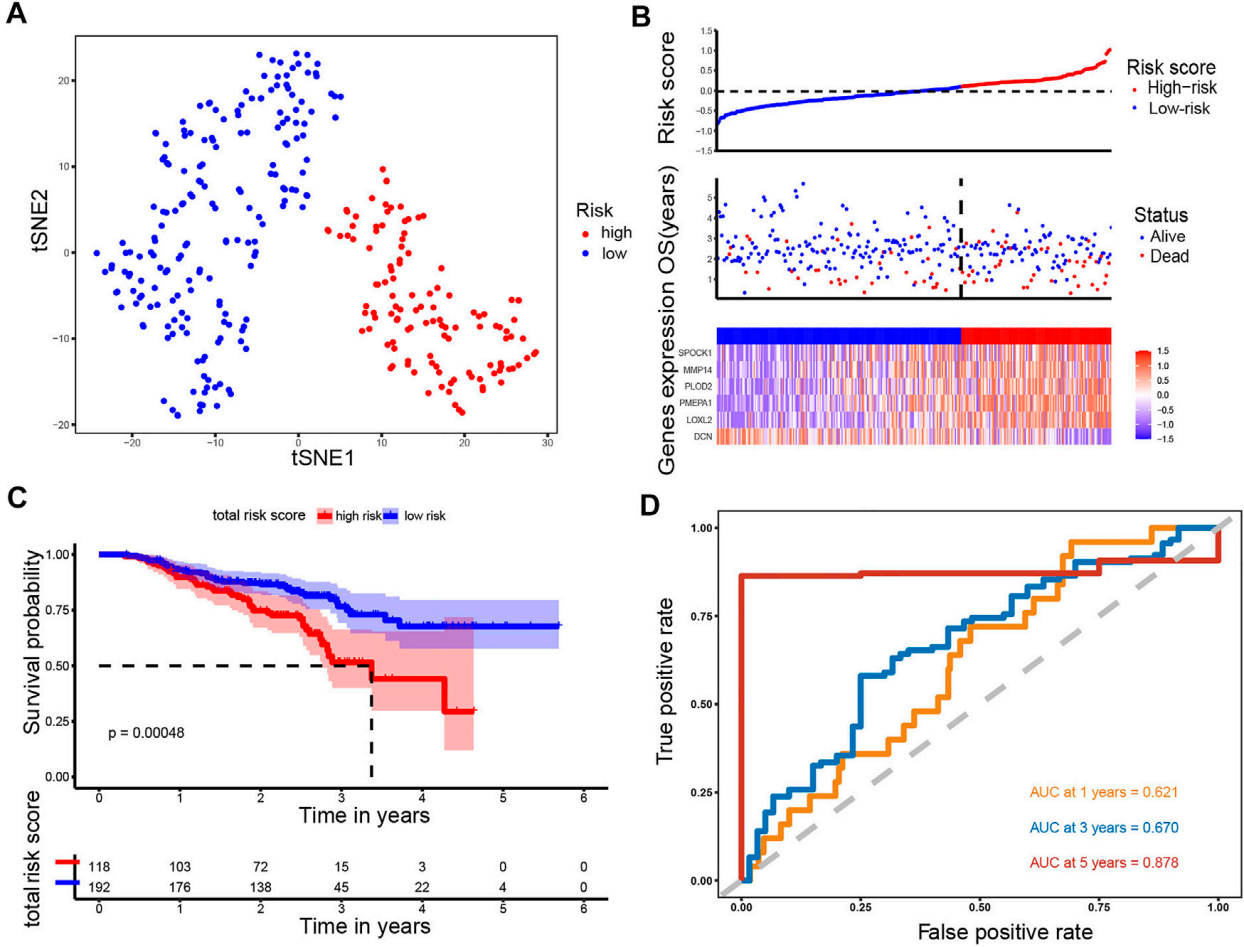
Analysis of the prognostic model in the test set. **(A)** t-SNE was used to evaluate whether the samples could be grouped correctly based on the ERGs risk score. **(B)** Heatmap showing the expression of six crucial genes in high- and low-risk groups and the distribution of risk scores and survival status of the LUAD patients with increasing risk score. **(C)** KM survival analysis between the high- and low-risk groups. **(D)** ROC curves analysis of the ERGs on OS at 1 year, 3 years, and 5 years.

Using the same formula and the cut-off value described above, patients in the GSE72094 cohort (N = 310) were stratified into the high-risk group (N = 118) and low-risk group (N = 192). Likewise, t-SNE analysis in the GSE72094 cohort confirmed that the patients in different risk groups were distributed into two directions (Figure 5A). The risk scores rank distribution, survival status and expression patterns of the 6 ERGs in the LUAD patients were shown in Figure 5B. In line with the results from the TCGA dataset, patients in the high-risk group showed significantly worse OS as opposed to those in the low-risk group (*p* = 0.00048; Figure 5C). ROC analysis demonstrated that the ERGs exhibited precise predictive capacity. AUCs at 1-, 3- and 5-year OS was 0.621, 0.670, and 0.878, respectively (Figure 5D). Together, these results indicated that the established prognostic model was capable of universal application.

### Construction and evaluation of the nomogram

**FIGURE 6:**
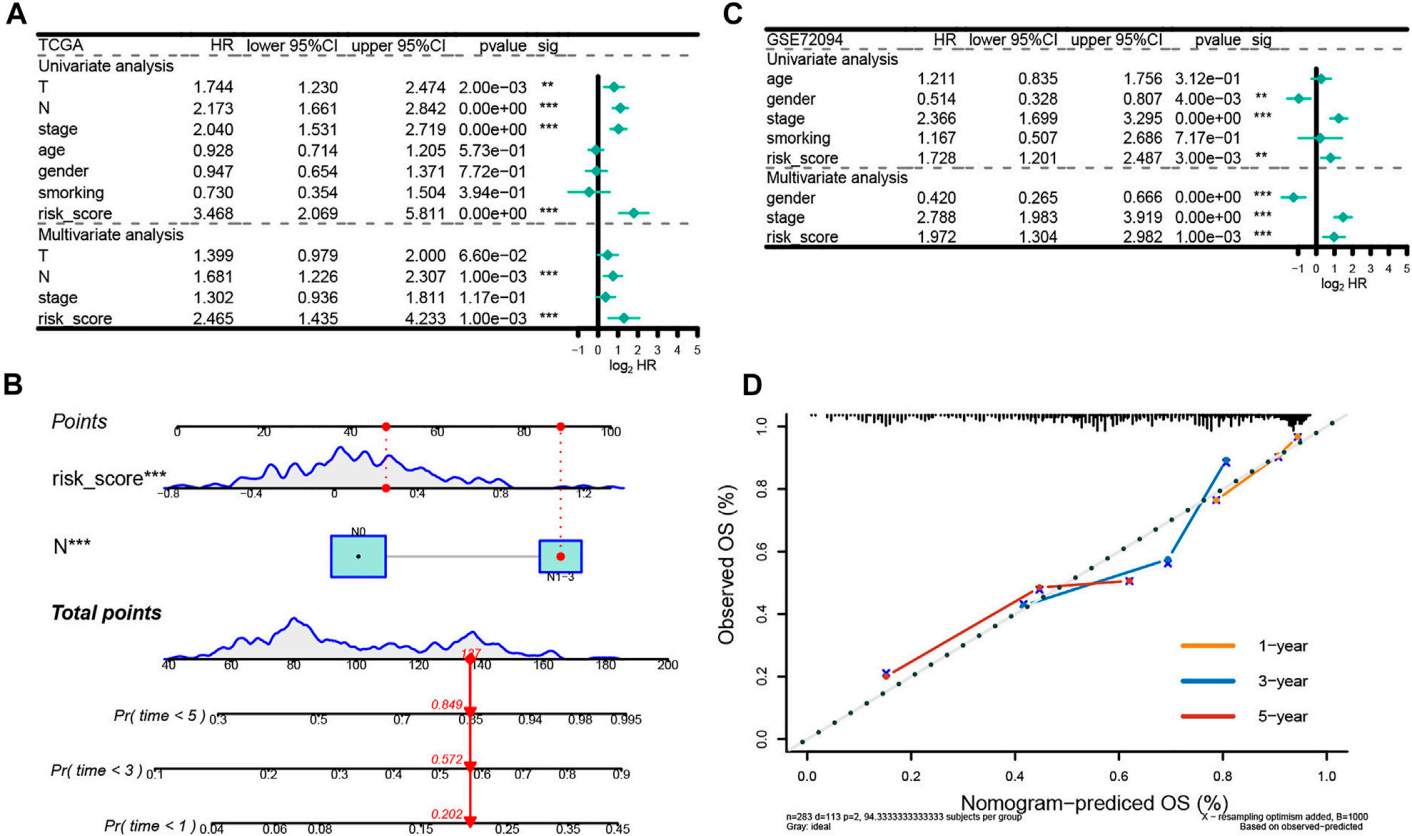
Independent prognostic analysis of the prognostic model. **(A)** Independent prognostic factors in the training set. **(B)** Independent prognostic factors in the test set. **(C)** The nomogram to predict overall survival was created based on independent prognostic factors. The 1-year, 3-year, and 5-year survival rate is predicted according to the total score. **(D)** The correction curve based on the prediction model.

To further evaluate the effect of the ERGs in predicting prognosis, we employed univariate and multivariate Cox regression analyses. Results from the univariate Cox regression analysis showed that T, N, stage and risk score were all significantly associated with OS, while the multivariate Cox regression analysis indicated that N stage and risk score were correlated with OS in patients with LUAD (HR:1.681, 95% CI (1.226–2.307), *p* < 0.001; HR: 1.302, 95% CI (0.936–1.811), *p* < 0.001) (Figure 6A). Moreover, the regression analyses in the GSE72094 indicated that the pathological stage and risk score were independent prognostic factors for OS (Figure 6B). These data demonstrated that the signature was an independent risk factor for survival in patients with LUAD.

We then developed a nomogram premised on the results from the multivariate Cox regression including the N stage and risk score, to predict 1-year, 3-year and 5-year OS, which contributed to defining higher risk scores (range 0–100 points) for the worse OS (Figure 6C). Each variable was allocated a score on the point scale. After summing up the points, the estimation of the survival likelihood was achieved by drawing a vertical line between the total points axis and the survival probability axes. Consistent with our previous findings, the nomogram illustrated the risk score as the prevailing contribution to prognosis compared with conventional clinical characteristics. In addition, the C-index was 0.715,896 and the calibration curves demonstrated that the predicted survival probability were highly consistent with the actual one (Keynesian cross) for 1-, 3-, and 5-year OS, which showed that this nomogram had great prediction performance (Figure 6D).

### Gene set enrichment analyses

**FIGURE 7:**
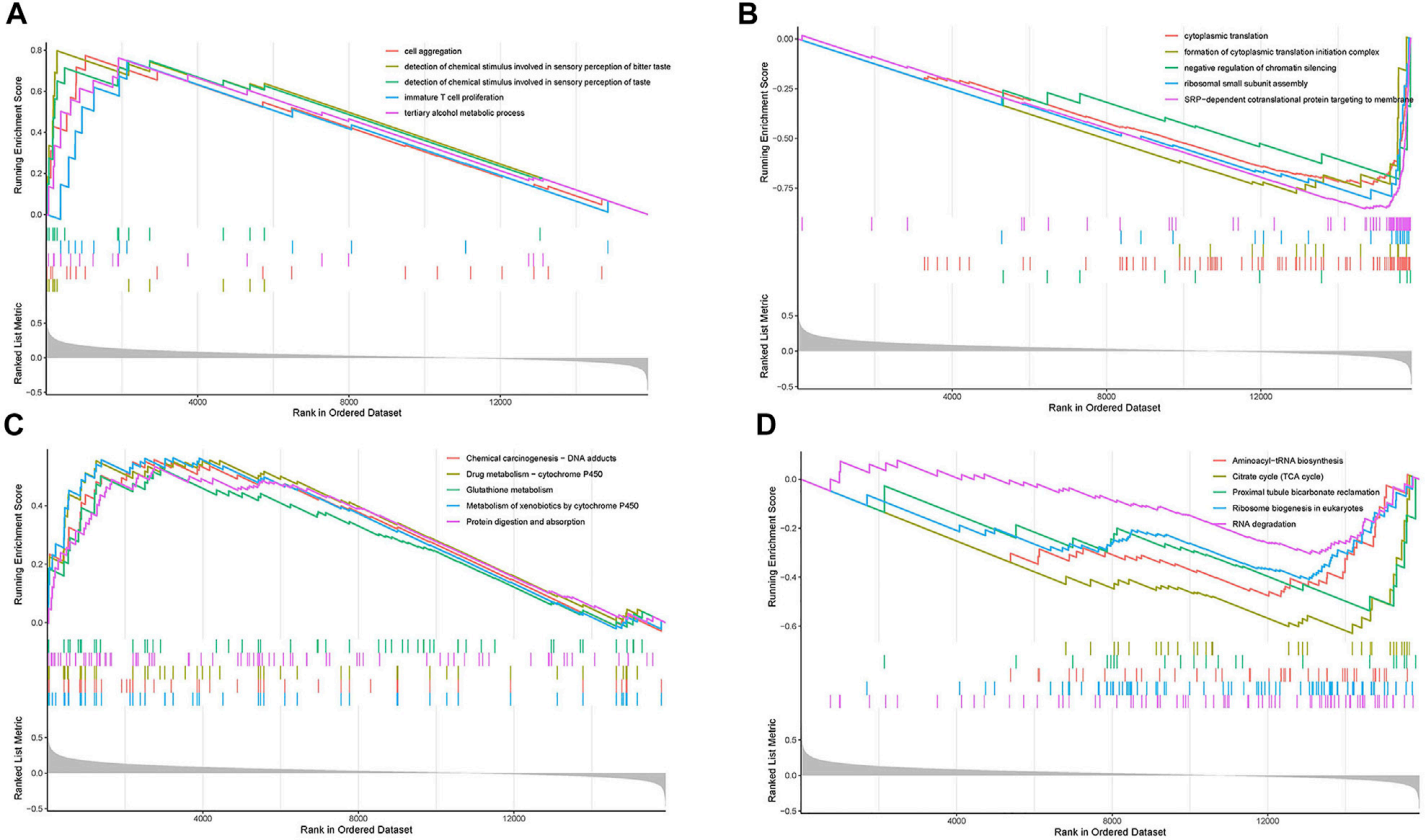
Gene set enrichment analysis (GSEA). **(A)** The top five enriched GO pathways in the high-risk group. **(B)** The top five enriched GO pathways in the low-risk group. **(C)** The top five enriched KEGG pathways in the high-risk group. **(D)** The top five enriched KEGG pathways in the low-risk group.

To evaluate the potential mechanisms of the ERGs in the high and low risk groups, we performed GSEA to identify GO terms and KEGG pathways in the TCGA cohort (*p* < 0.05). The GO analysis showed that the most concentrated biological processes in the high-risk group included cell aggregation, detection of chemical stimulus involved in sensory perception of bitter, detection of chemical stimulus involved in sensory perception of taste, immature T cell proliferation, and tertiary alcohol metabolic process (Figure 7A), while the most enriched biological processes in the low-risk group were cytoplasmic translation, formation of cytoplasmic translation initiation complex, negative regulation of chromatin silencing, ribosomal small subunit assembly and SRP-dependent co-translational protein targeting to membrane (Figure 7B). On the other hand, the KEGG analysis showed that the genes in the high-risk group were mainly enriched in pathways such as chemical carcinogenesis-DNA adducts, drug metabolism-cytochrome P450, glutathione metabolism, metabolism of xenobiotics by cytochrome P450 as well as protein digestion and absorption (Figure 7C). The genes in the low-risk group were mainly enriched in aminoacyl-tRNA biosynthesis, citrate cycle (TCA cycle), proximal tubule bicarbonate reclamation, ribosome biogenesis in eukaryotes and RNA degradation (Figure 7D). Moreover, most of the GO terms and KEGG pathways enriched in our analysis were closely associated with the occurrence and development of LUAD, which indicated that the ERGs may play a key role in cancer development and revealed potential pathways that could serve as therapeutic targets in LUAD.

### Analysis of the mutational landscape based on ERGS

**FIGURE 8:**
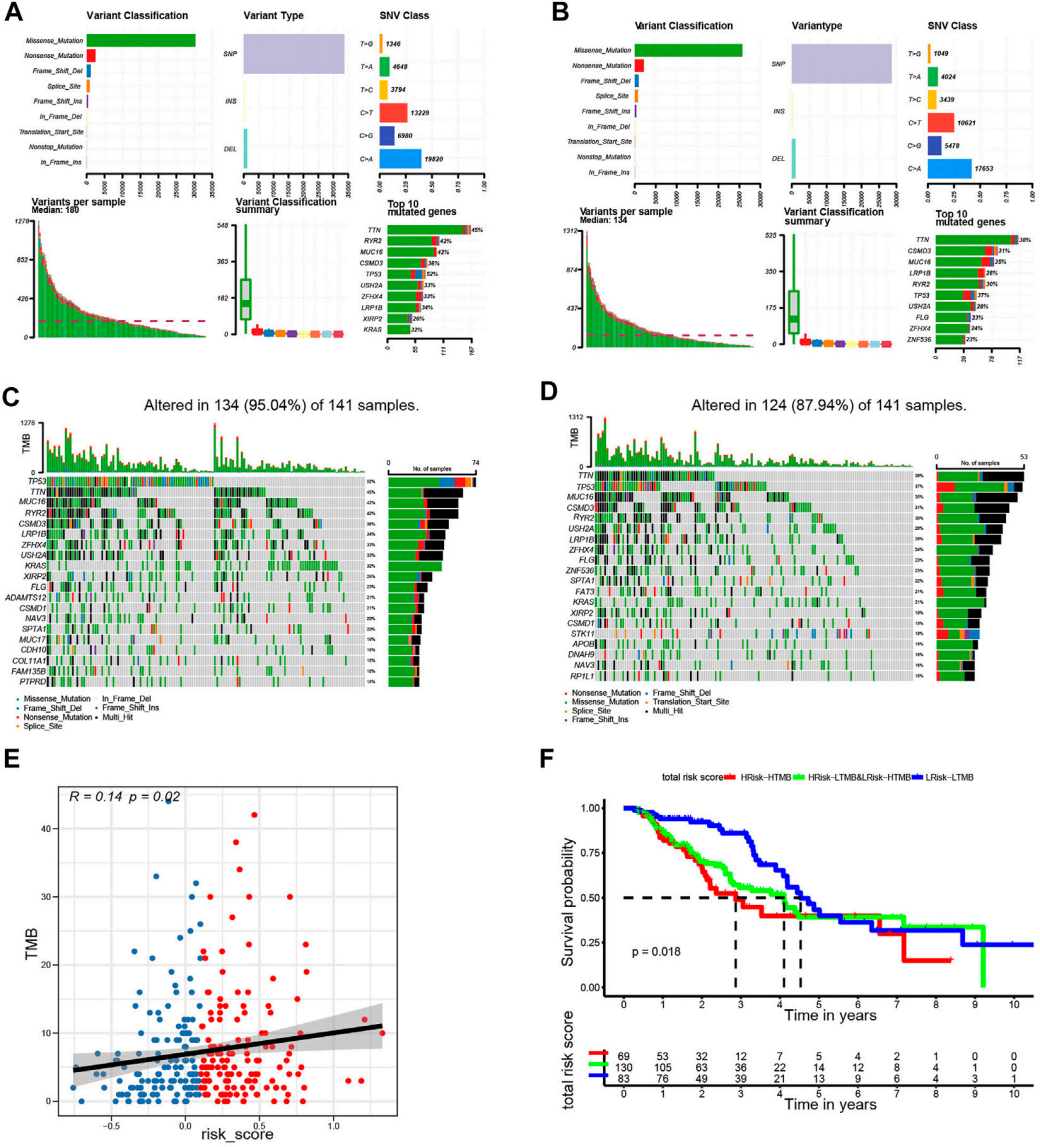
Landscape of mutation profiles in the low- and high-risk groups. **(A)** Overview of mutation types in the high-risk group. **(B)** Overview of mutation types in the low-risk group. **(C)** Waterfall Plot of the top 20 genes with the most mutations in the high-risk group. **(D)** Waterfall Plot of the top 20 genes with the most mutations in the low-risk group. **(E)** The correlation between risk score and TMB. **(F)** Kaplan-Meier curves for patients stratified by risk score combined with TMB.

In further characterize the high- and low-risk groups at the genomic level, the mutation status of both groups was analyzed. Results showed that missense mutations were the most prevalent of all mutation types followed by nonsense and frameshift deletions in both groups. The main variant was single nucleotide polymorphism (SNP), in which the single-nucleotide variant (SNV) with T>G was the most frequent (Figure 8A,B). The top 20 most common mutated genes in the high- and low-risk groups ranked based on percentages are shown in Figures 8C,D. In the low-risk group, 86.93% of the samples carried mutations. The top 10 mutated genes were TTN, CSMD3, MUC16, LRP18, RYR2, USH2A, TP53, FLG, ZFHX4, and ZNF536. The mutation frequency was higher in the high-risk group (94.74%) compared with the low-risk group. The top 10 factors linked to mutations were TTN, MUC16, RYR2, CSMD3, TP53, USH2A, ZFHX4, LRP1B, KRAS, and FLG. Subsequently, the relationship between the TMB and the risk score was examined. It was found that the risk score was positively correlated with TMB (R = 0.14, *p* < 0.05, Figure 8E), suggesting that the risk score could be an accurate indicator of the characteristics and performance of TMB in tumors. Further analysis revealed that the TMB status did not affect the survival outcome predicted using the risk score. The KM curve showed that the survival outcome was different between the subgroups (high-risk and high TMB vs. high-risk and low TMB, low-risk and high TMB vs. low-risk and low TMB, *p* = 0.018; Figure 8F). The low-risk and low TMB subgroup showed the highest overall survival rate, whereas the high TMB and high-risk group had the lowest survival rate.

### Evaluation of immune infiltration status

**FIGURE 9:**
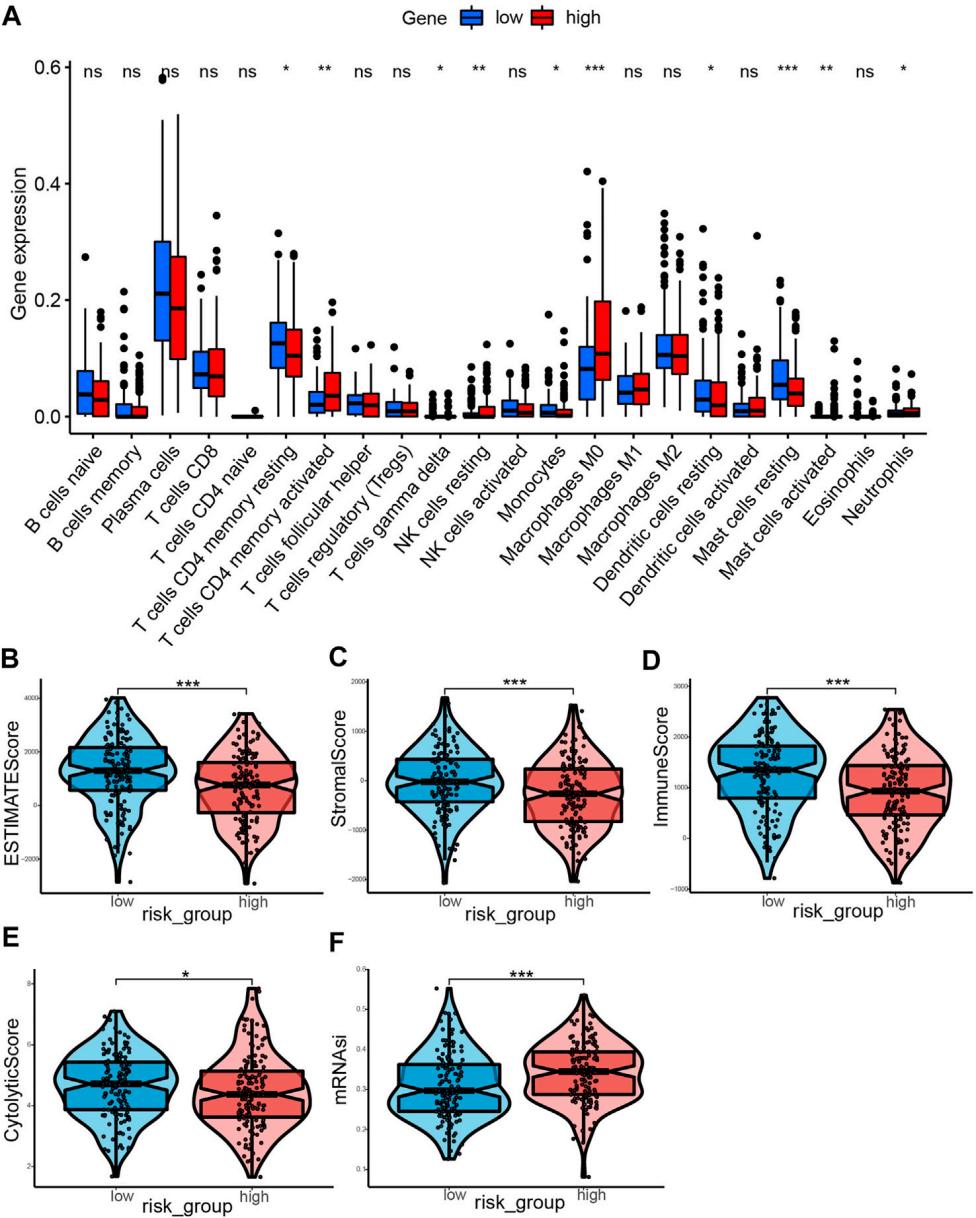
Landscape of immune Infiltration profiles in the low- and high-risk groups. **(A)** The ratio differentiation of 22 kinds of immune cells between the low- and high-risk group, and the Wilcoxon rank sum was used for the significance test. **(B–F)** The violin plot showed the differences of ESTIMATE score, stromal score, immune score, CYT score and mRNAsi score between the low- and high-risk groups. *p < 0.05, **p < 0.01, ***p < 0.001.

The CIBERSORT tool was utilized to calculate the infiltration degree of 22 immune cells (Figure 9A). Results showed that the resting memory CD4 T cells, gamma delta T cells, monocytes, resting mast cells, and resting dendritic cells were higher in low-risk group compared with the high-risk group. On the contrary, the high-risk group had high proportions of activated memory CD4 T cells, resting NK cells, M0 macrophages, activated mast cells and neutrophils compared with the low-risk group. Subsequently, we explored the relationship between immune cells and the risk score. It was found that the risk score was positively associated with the level of activated memory CD4 T cells, M0 macrophages, activated dendritic cells, activated mast cells, and resting NK cells (*p* < 0.05, Supplementary Figure S1A–E), however, it was negatively correlated with the level of T cells CD4 memory resting, macrophages M2, eosinophils, mast cells resting, and dendritic cells resting (*p* < 0.05, Supplementary Figure S1F–J). Having identified the effects of ERGs on the regulation of TME remodeling, we further investigated whether ERGs expression levels were correlated with the abundance of immune cells. It was identified that these six prognostic genes were differentially correlated with immune cell infiltration (Supplementary Figure S2). Furthermore, the estimate score, immune core, and stromal score were higher in the low-risk group compared with the high-risk group (*p* < 0.001, Figures 9B–D), suggesting that the infiltration levels of immune and stromal cells were higher in the low-risk group. In comparison, the high-risk group had low CYT score (*p* < 0.05) and high mRNAsi score (*p* < 0.001, Figures 9E,F). This demonstrated that patients in the high-risk group had lower antitumor immunity and higher neoplastic stemness. Consequently, tumor cells in these patients had stronger self-renewal, differentiation, and proliferation ability, which may explain their worse OS(Wang et al., 2021b).

### Assessment of chemotherapy efficacy

**FIGURE 10:**
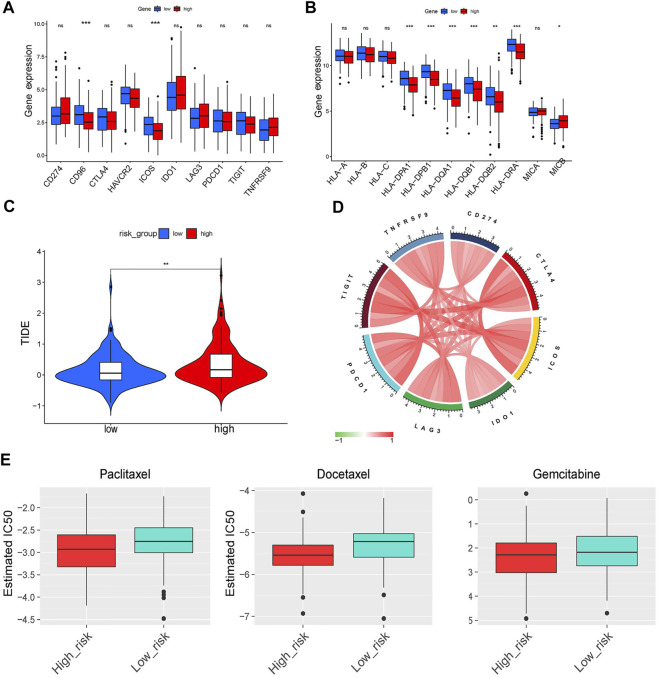
Immune checkpoints analysis and evaluation of response to ICI therapy and chemotherapy. **(A,B)** The differentiation of immune checkpoints between the low- and high-risk group. **(C)** The comparison of TIDE score between the low- and high-risk group. **(D)** Circos plot showing the interconnectivity among ERGS genes. The thickness and color of the ribbons depend on the correlation between the signature gene expression. **(E)** The sensitivity to paclitaxel, docetaxel and gemcitabine of patients with LUAD.

In subsequent analyses, we further explored the association between the ERGS and efficacy of chemotherapy in LUAD. Results showed that patients in the low-risk group had significantly lower IC50 values and were more sensitive to paclitaxel (p < 0.0001), docetaxel (p < 0.0001), and gemcitabine (p < 0.05) compared with those in the low-risk group, which suggested that the constructed model could effectively predict efficacy and sensitivity to chemotherapy (Figure 10E).

### Validation of gene expression

**FIGURE 11:**
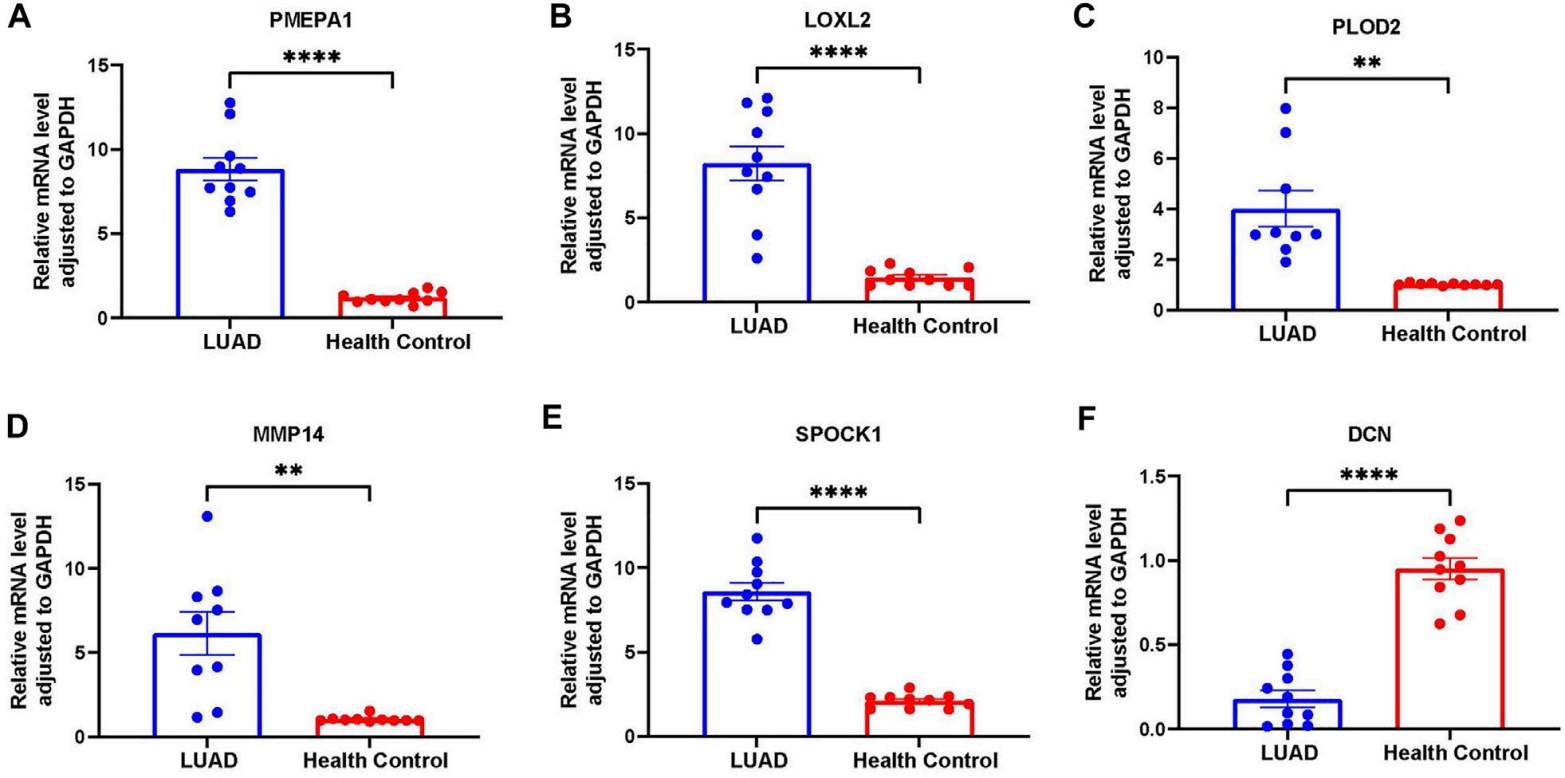
Further verification of the overexpression of six genes in RT-qPCR analysis. **(A)** PMEPA1 expression level in LUAD and health control tissues. **(B)** LOXL2 expression level in LUAD and health control tissues. **(C)** PLOD2 expression level in LUAD and health control tissues. **(D)** MMP14 expression level in LUAD and health control tissues. **(E)** SPOCK1 expression level in LUAD and health control tissues. **(F)** DCN expression level in LUAD and health control tissues.

To validate the expression profile of ERGs in LUAD patients, clinical specimens were collected from LUAD patients, together with adjacent normal tissue. These specimens were analyzed using RT-qPCR. It was found that LOXL2, PLOD2, MMP14 and SPOCK1 were upregulated in tumor samples, whereas DCN was downregulated in tumor specimens (Figure 11).

### Validation of protein expression

**FIGURE 12:**
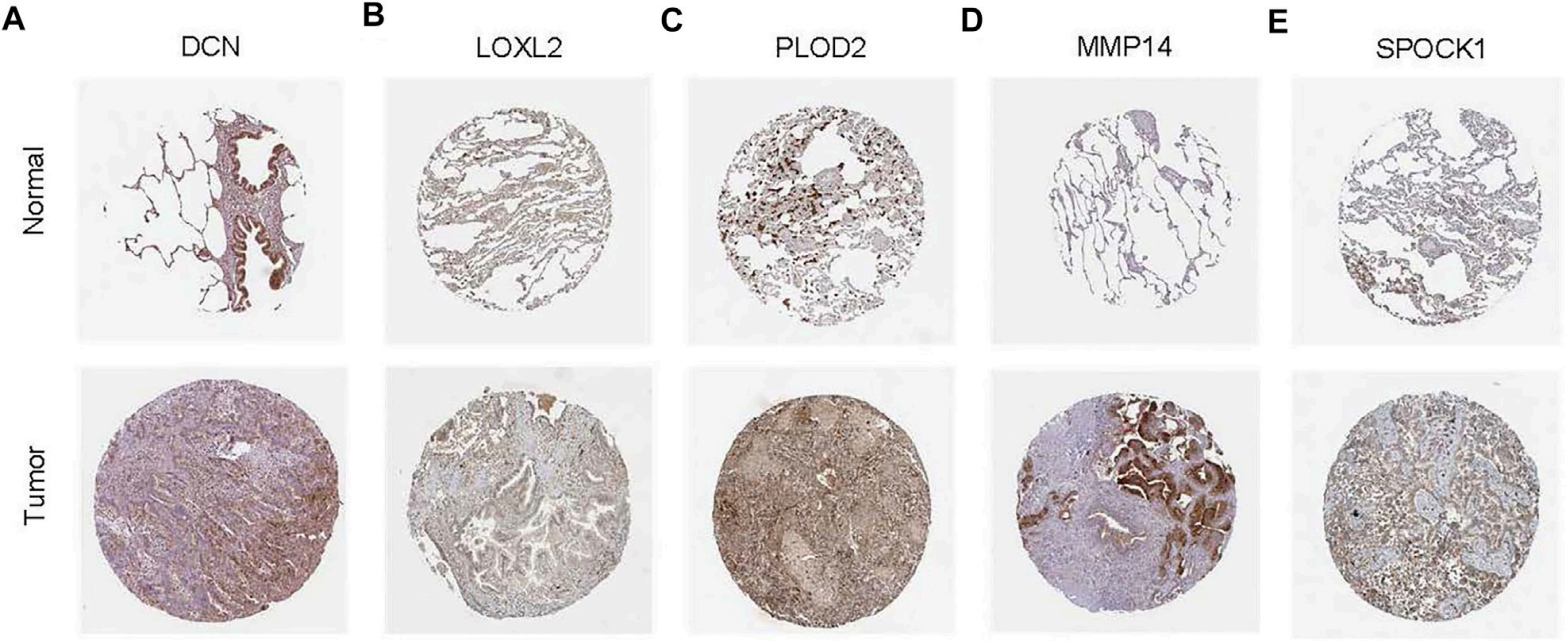
Representative immunohistochemical stains of the five prognostic genes in the HPA database. **(A)** Expression of DCN protein in LUAD and normal control samples. **(B)** Expression of LOXL2 protein in LUAD and normal control samples. **(C)** Expression of PLOD2 protein in LUAD and normal control samples. **(D)** Expression of MMP14 protein in LUAD and normal control samples. **(E)** Expression of SPOCK1 protein in LUAD and normal control samples.

The HPA is a public database with millions of immunohistochemical images and is used by researchers to compare protein expression patterns between normal and tumor tissues. Because the lung cancer data were not classified according to histological type in HPA, we analyzed the IHC staining of six ERGs in lung cancer to verify their expression levels. Notably, only protein expression staining images of five genes (DCN, LOXL2, PLOD2, MMP14, and SPOCK1) were found in HPA. Moreover, the results showed that the expression levels of LOXL2, PLOD2, MMP14 and SPOCK1 was higher whereas that of DCN was lower in tumor tissues compared with normal tissues (Figure 12).

## Discussion

The pathologic stage is a critical marker used for prediction of the prognosis of LUAD in routine clinical practice. However, the progression of LUAD is highly heterogenous in terms of genetic and epigenetic presentations (Lu et al., 2021). For this reason, patients with the same stage of disease may have different clinical outcomes (Yao et al., 2021). Accurate prognosis analysis is a critical factor of precision medicine in stratifying risks and developing an optimal management plan. A growing body of research has revealed that EMT process regulate several aspects of cancer cells including invasion, metastasis, refractory responses to chemotherapy and immunotherapy, immunosuppression, and acquisition of stem cell-like properties. Studies have also reported that the EMT process has been linked to metastasis and treatment resistance in LUAD (Wang et al., 2021a). Therefore, we constructed and validated a comprehensive signature based on EMT-related genes to predict the prognosis of LUAD patients using GEO and TCGA dataset.

The proposed signature consisted of six genes, DCN, PMEPA1, LOXL2, PLOD2, MMP14, and SPOCK1. Among them, PMEPA1, LOXL2, PLOD2, MMP14, and SPOCK1 were associated with poor outcomes whereas DCN was correlated with good prognosis of LUAD patients. PMEPA1 has been shown to induce tumorigenesis by interfering with several signaling cascades such as mutated p53, Hippo signaling, Wnt, and EGF (Qiu et al., 2021). It has also been reported to promote malignant behavior and enhance tumorigenic ability by activating MAPK/JNK signaling pathways (Tan et al., 2021). LOXL2 regulates collagen cross-linking and deposition in primary tumor tissue. In previous studies, it was found to promote tumor cell survival and development of drug resistance, regulate cell adhesion, motility, and invasion. Upregulation of LOXL2 enhanced the invasion and metastasis of lung cancer (Peng et al., 2017). Moreover, LOXL2 has been incorporated in prognostic models to predict late recurrence in LUAD patients (Zhao et al., 2021). PLOD2 is one of the members of the PLOD family that encodes the lysyl hydroxylase 2. It modulates collagen cross-link formation in the extracellular matrix. In previous studies, PLOD2 was reported to promote tumor metastasis by inducing collagen cross-linking (Yamauchi and Sricholpech, 2012; Chen et al., 2015). A bioinformatics study showed that the PLOD family members can be novel biomarkers for predicting LUAD prognosis (Meng et al., 2021). Being a member of the first membrane matrix metalloproteinases (MMPS), MMP14 has been shown to promote extracellular matrix (ECM) degradation to accelerate tumor cell migration, inflammation, invasion, angiogenesis and metastasis. Its expression was found to be increased in colorectal cancer, lung cancer, and nasopharyngeal carcinoma, leading to enhanced tumor progression (Yan et al., 2015; Stawowczyk et al., 2017; Cui et al., 2019). SPOCK1 encodes a matricellular glycoprotein belonging to a new Ca (2+)-binding proteoglycan family, which promotes cell proliferation, adherence, and migration (Bradshaw and Sage, 2001). High SPOCK1 expression has been associated with increased invasiveness, growth, and metastatic potential (Wang et al., 2018). In lung cancer, high expression of SPOCK1 correlated with poor prognosis. SPOCK1 is a novel TGF-β-targeted gene that regulates lung cancer epithelial cells (Basu et al., 2018). As a protective factor, DCN has been suggested to block receptor tyrosine kinases thereby suppress lung cancer progression (Horváth et al., 2014). This view is consistent with that of previous articles.

The immune components of the TME can promote or inhibit or tumor progression (Papait et al., 2020). It is important to understand the underlying mechanisms that are involved between EMT and the TME. In this study, the TME was assessed using CIBERSORT and the ESTIMATE algorithm. The relationship between the risk score and immune-infiltrating cells was evaluated. Results showed that tumors in the high-risk group showed higher infiltration of immunosuppressive cells such as macrophages, neutrophils, and mast cells compared with tumors in the low-risk group. This indicated that the EMT process may protect tumors from the intrinsic anti-tumor immune response by creating an immunosuppressive microenvironment. Therefore, the EMT process may explain the poor prognosis of the high-risk group. Previously, it was found that patients with high levels of M0 macrophages had enhanced EMT process, hence poor prognosis (Dong et al., 2021). Our findings corroborate the results that mast cell-derived extracellular vesicles induced EMT by signaling cascades (Yin et al., 2020). Activated memory CD4^+^ T cells were also highly expressed in the high-risk group. We speculate that cytokines released by activated T cells, such as IL-6, TNF, and TGFβ, can promote EMT development (Cohen et al., 2015). It was reported that CD4+T cells can induce EMT-like features in clear cell renal carcinoma cells by secreting IL-6 (Chen et al., 2017).

In this study, the low-risk subgroup showed higher immunoactivity of immune checkpoint molecules, immune score, and cytolystic activity. The higher mRNAsi was found in the high-risk group, which may explain why EMT results in poor prognosis. The tumor immune escape decreases the expression of HLA which enables tumor cells to avoid the cytolysis function of T cells (Garcia-Lora et al., 2003). We found that expression of HLA genes was significantly downregulated in the high-risk group, which indicated that immune escape occurred in the high-risk group. Previous study also reported that EMT inhibits the formation of immune synapses between cancer cells and T cells leading the immune escape (Akalay et al., 2013). Based on the above results, we speculated that there might be significant differences in the efficacy of immunotherapy between the two groups. Therefore, we examined the response to ICI therapy using the TIDE algorithm. Results showed that patients in the low-risk group were more likely to respond to immunotherapy.

Further analysis showed that the six ERGs signature predicted the responses to several common chemotherapeutic agents. Particularly, the low-risk group was more sensitive to chemotherapy drugs such as, paclitaxel, docetaxel, and gemcitabine compared with the high-risk group. The lower response in the high-risk group may be due to EMT-induced drug resistance. Paclitaxel and docetaxel, two chemotherapy drugs belonging to the taxene family, have been shown to induce cell cycle arrest in cancer cells by preventing microtubule depolymerization. EMT-induced invasive behavior of cancer cells can cause tolerance towards Paclitaxel and docetaxel. Upstream mediators of EMT, such as ZEb1/2, TGF-β, and microRNA regulate response of cancer cells to Paclitaxel and docetaxel (Ashrafizadeh et al., 2021). During EMT process, the conversion of E-cadherin to N-cadherin reduces the expression of human balanced nucleoside transporter 1 (hENT1), a drug carrier for gemcitabine membrane transport in cancer cells, which triggers gemcitabine resistance in cancer cells (Weadick et al., 2021). In general, we found that patients with low-risk may benefit more from immunotherapy and chemotherapy compared with those with high-risk. This implies that more studies are needed to develop new treatment strategies or multi-drug combinations to improve prognosis of high-risk patients.

Overall, the proposed ERGS signature showed good performance in predicting the prognosis of LUAD. Our results highlight the need to investigate the role of EMT in the progression of LUAD. The constructed prognostic model can also be used to evaluate the tumor immune microenvironment, guide application of individualized therapy, and facilitate the development of targeted therapy. Nevertheless, this study has some limitations. First, this was a retrospective study and independent prospective cohorts are needed to validate the prognostic model developed in the study. The value of six genes as potential targets also needs further investigations. Second, this risk model is highly relied on public databases. As a result of the clinical information downloaded from TCGA and GEO databases is limited or incomplete data, potential prognostic factors, such as personal clinical history and treatment intervention, are missing in our nomogram. It is not clear How the environmental factors such as smoking and exposure to certain toxins might have influence the identified gene signatures. Further investigations need to be undertaken in future clinical researches.

## Conclusion

This study constructed a novel prognostic model based on 6 EMT-related genes. The model was established in the training cohort and validated using an external validation cohort, RT-PCR tests, and IHC assays. The results showed that the model was a robust biomarker for predicting the OS in LUAD patients. Furthermore, according to the TME analysis and evaluation of chemotherapy efficacy, the features indirectly demonstrated that patients in the low-risk group based the model had a higher likelihood to benefit from immune therapy and chemotherapy. This study provides new reference findings for further exploration of the mechanisms of EMT and tumor immunity. It also provides insights to guide personalized treatment of LUAD patients.

## Data Availability

The datasets presented in this study can be found in online repositories. The names of the repository/repositories and accession number(s) can be found in the article/Supplementary Material.
