# Supplementary material for: Identifying the EMT-related signature to stratify prognosis and evaluate the tumor microenvironment in lung adenocarcinoma
Source: Front Genet. 2022 Sep 16;13:1008416. doi: 10.3389/fgene.2022.1008416 (PMC9523218; doi:10.3389/fgene.2022.1008416)
Supplement: Supplementary file 1 [file DataSheet1.ZIP › Figure S1-2.docx]

Supplementary Material

## Supplementary Figures

##
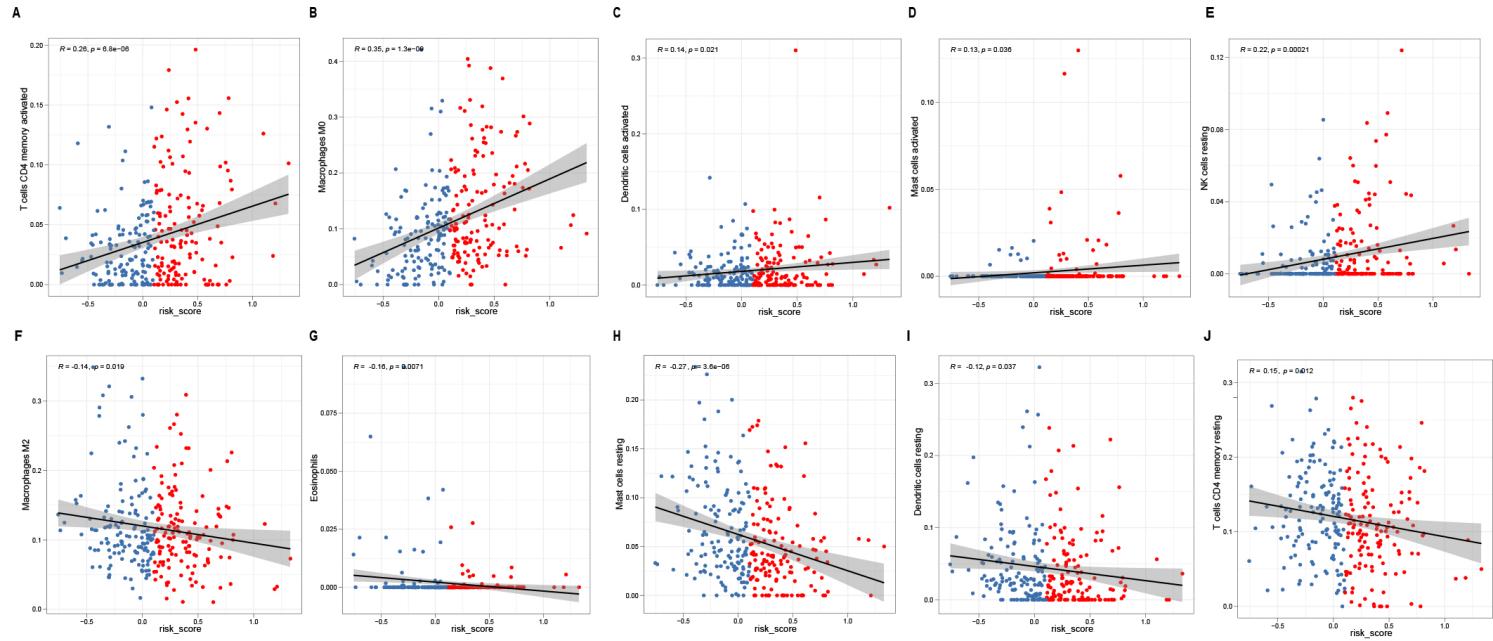
**Supplementary Figure 1.** Correlation of immune cell subtypes with six-gene signature risk score in the training cohort. (A) Activated memory CD4 T cells. (B) M0 macrophages. (C) Activated dendritic cells. (D) Activated mast cells. (E) Resting NK cells. (F) Macrophages M2. (G) Eosinophils. (H) Mast cells resting. (I) Dendritic cells resting. (J) T cells CD4 memory resting.


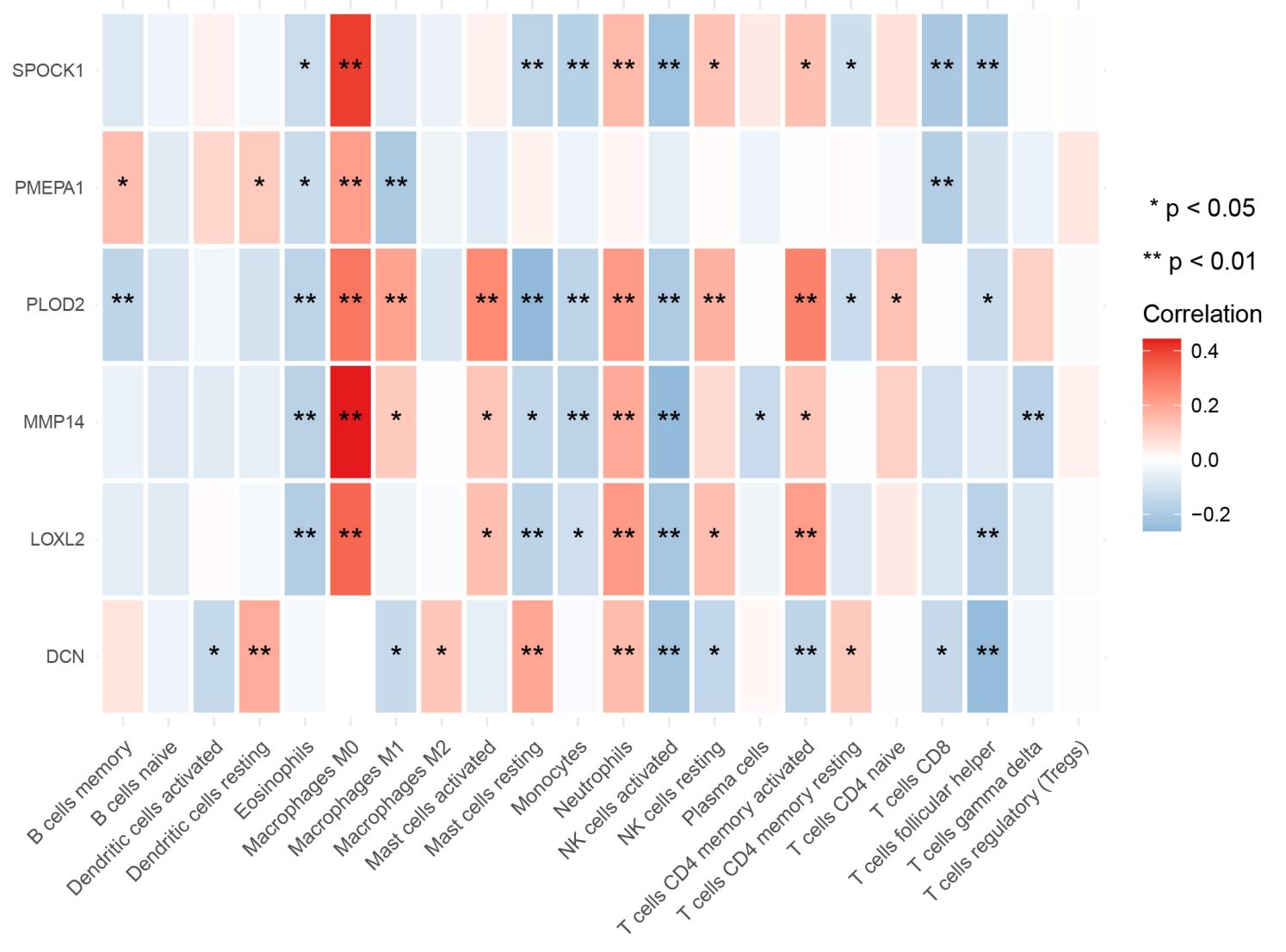


**Supplementary Figure 2.** Correlation of immune cell subtypes with six prognostic genes.
