# Supplementary figures and images for: Identifying the EMT-related signature to stratify prognosis and evaluate the tumor microenvironment in lung adenocarcinoma
Source: Front Genet. 2022 Sep 16;13:1008416. doi: 10.3389/fgene.2022.1008416 (PMC9523218; doi:10.3389/fgene.2022.1008416)

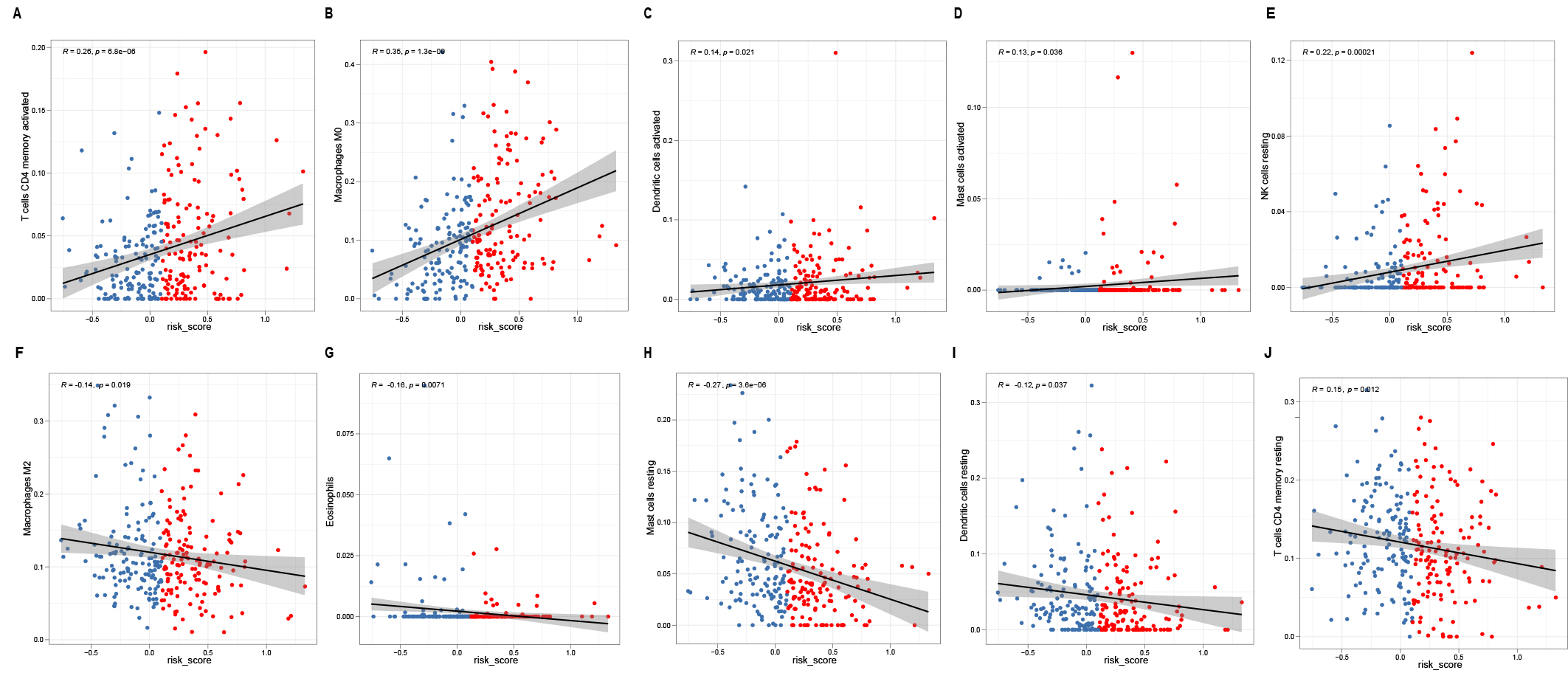

Supplement: Supplementary file 1 [file DataSheet1.ZIP › FigureS1.jpg]

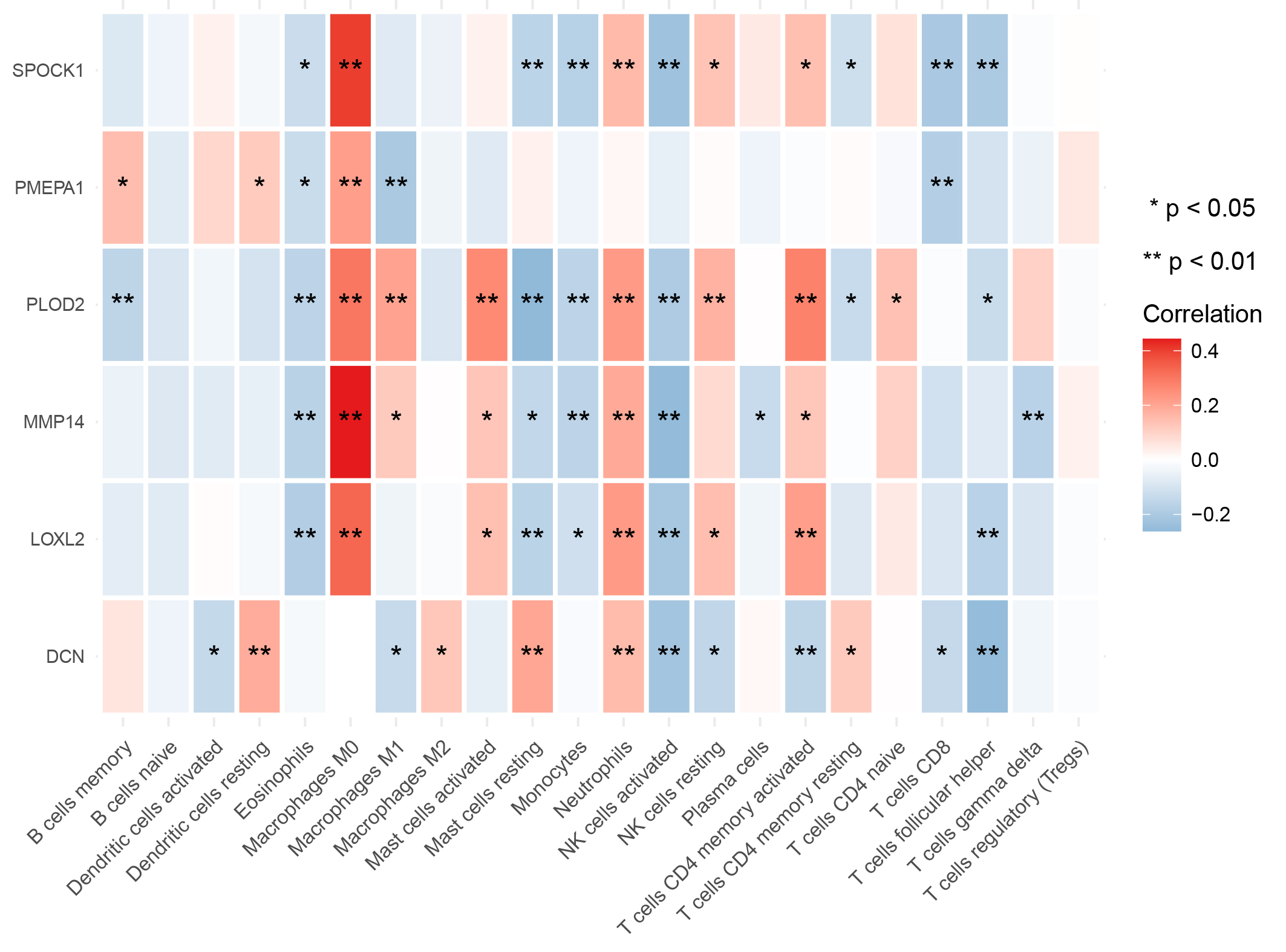

Supplement: Supplementary file 1 [file DataSheet1.ZIP › FigureS2.jpg]
